# Outcomes after EVAR in females are similar to males

**DOI:** 10.1186/s12872-021-02114-2

**Published:** 2021-06-15

**Authors:** Naim Boran Tumer, Goktan Askin, Bekir Bogachan Akkaya, Isa Civelek, Ertekin Utku Unal, Hakki Zafer Iscan

**Affiliations:** 1 Department of Cardiovascular Surgery, Ankara City Hospital, Ankara, Turkey; 2 Department of Cardiovascular Surgery, Hitit University Erol Olcok Training and Research Hospital, Çorum, Turkey

**Keywords:** Female gender, EVAR, Elective iAAA

## Abstract

**Introduction:**

Women are less likely to develop infrarenal abdominal aortic aneurysm; however, when they do, it is almost always associated with challenging anatomy, more rapid aneurysmal growth rate and earlier rupture. Women generally have poorer outcomes following open aneurysm repair; and in this respect, the present study aims to evaluate if it is so after endovascular repair.

**Methods:**

A retrospective analysis of our database was performed for patients underwent endovascular aneurysm repair (EVAR) between January 2013–March 2020. 249 elective EVAR patients were evaluated. Patients were categorized according to gender and 26 patients (10.4%) were female. Demographics and pre-peri-postoperative findings were compared. Propensity score matching (ratio 1:1) was performed to reduce selection bias.

**Results:**

In the overall unmatched cohort, female population had more diabetes mellitus (*p* = 0.016) and hypertension (*p* = 0.005). However, coronary artery disease (*p* = 0.005) and coronary artery bypass grafting (*p* = 0.006) were more in male gender. Non-IFU implantation was higher in female group (38.5% vs. 11.5%, *p* = 0.025). After propensity matching, even though it was not statistically significant, early mortality for female gender was higher when compared to male gender (7.7% and 0%, respectively, *p* = 0.490). In the follow-up period, no difference in all-cause mortality, secondary interventions or complications have been observed between the genders.

**Conclusion:**

Challenging anatomy and subsequently treated patients outside IFU may be the reasons for higher morbidity and mortality in women. However, despite these factors female and male patients revealed equivalent early and late results.

## Introduction

Abdominal aortic aneurysm (AAA) prevalence according to the screening programs over 65 years old men is around 1.3–3.3% [1]. It is believed to reach about 15% over the age of 75 [2]. A recent systematic review of publications between 2000 and 2015 indicates that the pooled prevalence of AAA in women over 60 years was 0.7% [1, 3]. Even though the incidence and prevalence of AAA in female gender is only one quarter of males, the Society of Vascular Surgeons recommends population screening for AAA for men and women over 65 years at the current guideline for the first time, however ESVS does not [1, 4]. The current guidelines indicate intervention at a diameter over 5 cm for women where it is over 5.5 cm for men [1, 4]. Women usually present with smaller aneurysms while having greater growth rates and moreover, there are differences between genders at every stage of the disease, from epidemiology to pathophysiology and from morbidity to mortality [5].

In the last decades, successful early and satisfactory long-term results with previous generation endografts have been widely reported for endovascular aortic aneurysm repair (EVAR), offering a less invasive approach with lower morbidity and mortality when compared to open surgery [6–9]. Gender differences in vascular procedures always receive attention. Despite worse outcomes have been reported in women for open surgery, endovascular repair of infrarenal AAA have conflicting results [6]. Although it is less common in women, when there is such diagnosis, it is almost always associated with poor surgical outcomes, more rapid aneurysmal growth rate, and earlier rupture. One out of the many things that is not understood about women is the high morbidity mortality in aortic aneurysms.

In cross-sectional studies, since the number of cases is limited, interpretations were made on gender with reviews and meta-analyses; and these interpretations contained controversies due to heterogeneities [10]. This study aims to evaluate the outcomes of elective EVAR procedures in women with infrarenal AAA and to compare with male patients in our EVAR experience.

## Patients and methods

After Ankara City Hospital local ethics committee approval (E1-20-516), a retrospective analysis of our database for patients undergoing EVAR procedure was performed. Patients were eligible for inclusion in the current study if they underwent an elective EVAR from January 2013 to March 2020. A total of 249 elective EVAR patients were evaluated. Patients were divided into groups according to gender; and all demographics and pre-peri-postoperative findings were compared. Body surface area (BSA) was calculated according to Dubois Formula [11]. The aortic size index (ASI) was determined by the ratio of BSA to diameter of the aorta. Considering the study of Davies et al., those with ASI < 2.75 cm/m^2^ were considered to have low risk (annual risk approximately 4%), those with ASI between 2.75 and 4.25 cm/m^2^ were considered to have moderate risk (annual risk approximately 8%), and those with ASI above 4.25 cm/m^2^ were categorized to have high risk (annual risk approximately 20–25%) [12]. Furthermore, emergency cases, hybrid procedures and procedures with concomitant surgery were excluded. Early mortality was defined as 30 day and/or in-hospital mortality. The variables such as age, pre-operative comorbidities, hematocrit, serum creatinine, pre-operative electrocardiogram (ECG), echocardiography, AAA diameter and instructions for use (IFU) compatibility were evaluated. Coronary angiography was performed only for symptomatic patients. Others were evaluated with routine ECG and echocardiography. Despite the differences between the IFU criteria’s of the devices, proximal aneurysm neck diameter 18–32 mm, neck angulation < 60 degrees, infrarenal neck length > 10 mm, iliac diameter 8–22 mm, and distal fixation length > 15 mm accepted as positive for IFU criteria. Pre-operative cardiac and pulmonary comorbidity were recorded as either no abnormality or abnormality. Perioperatively, amount of contrast agent, duration of fluoroscopy, intensive care unit (ICU) time and length of hospital stay (LOS) were included in the evaluations. Technical success was the completion angiography without type I or III endoleak for every patient. The primary endpoints of the study were early (30-day) mortality and midterm mortality. Morbidities and secondary interventions performed after EVAR were the secondary endpoints.

### Statistical analysis

**Fig. 1:**
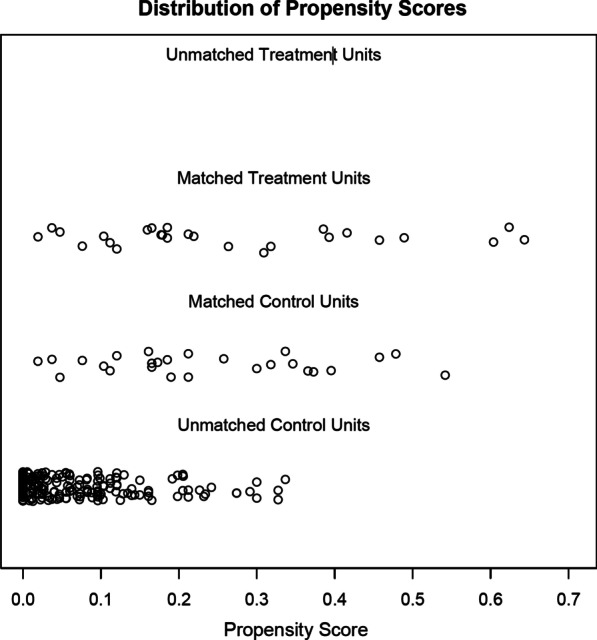
Jitter plot analysis showing distribution of propensity scores

**Fig. 2:**
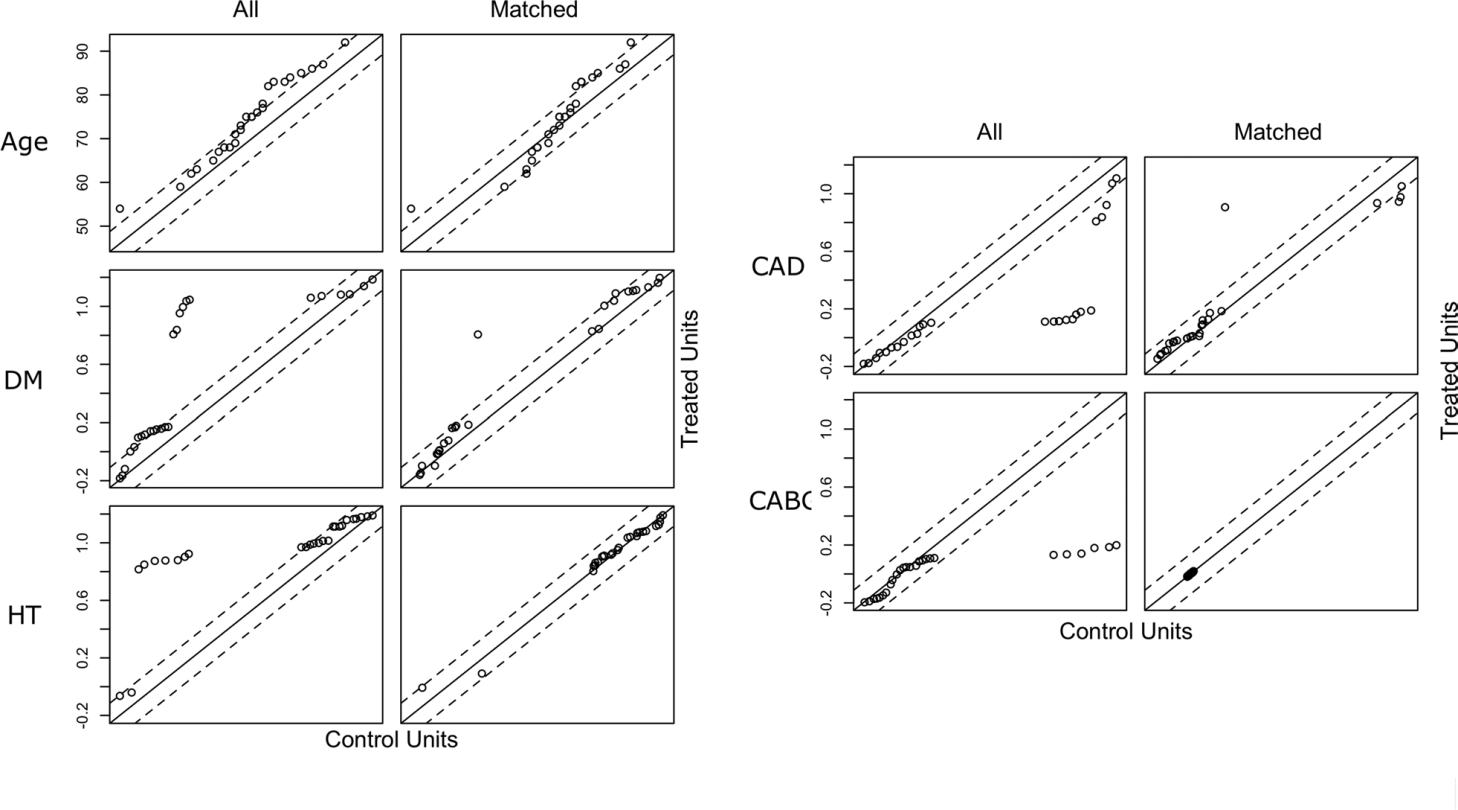
Plots showing balance on the covariates

The variables were investigated using visual (histograms, probability plots) and analytical methods (Kolmogorov–Smirnov/Shapiro–Wilk test) to determine the normality of their distribution. Normally distributed continuous variables were expressed as mean ± standard deviation (SD), or median values with an interquartile range if not normally distributed. Categorical variables were expressed as numbers and percentages. Demographic characteristics, perioperative variables, and calculated values were compared using the independent Samples t-test or the Mann–Whitney U-test for continuous variables, and the Chi-Square test or Fisher’s Exact Test for categorical variables. Propensity score matching (PSM) was performed by matching patients by gender, controlling for age and baseline comorbidities in a 1:1 ratio (R statistical software, ver 4.0.2). Nearest neighbor matching without replacement was performed on the propensity score for covariates such as age, diabetes, hypertension, coronary artery disease and coronary artery bypass grafting. Each female patient was paired with an available male patient that has the closest propensity score. Matching balance was assessed with standardized mean differences and jitter plot for all cohort and plots for each covariate (Table 1, Figs. 1, 2). A *p* value of < 0.05 was statistically significant, and all other statistical analyses were performed using the SPSS for Windows version 15.0 statistical software program (SPSS Inc., Chicago, IL, USA).

**Table 1:** Standardized mean differences for covariates before and after matching

Covariate	SMD before matching	SMD after matching
Age	4.6049	1.3462
DM	0.2194	0.0385
HT	0.2684	0.0000
CAD	0.2920	0.0385
CABG	0.2332	0.0000

*SMD* standardized mean difference, *DM*diabetes mellitus, *HT* hypertension, *CAD* coronary artery disease, *CABG* coronary artery bypass grafting

## Results

### Baseline characteristics and peri-operative details

In this retrospective study, 249 EVAR patients between 2013 and 2020 were analyzed. A total of 26 patients were female (10.4%) with mean age of 74.2 ± 9.5 years (median 75 years) and were older than the male population for unmatched groups (mean age 69.6 ± 7.6 years, median 69 years) (*p* = 0.014). Moreover, for the unmatched groups, diabetes and hypertension were found to be higher in female population. Whereas coronary artery disease and coronary artery bypass grafting was more common in male gender (Table 2). After matching, there were 26 female and 26 male patients. All baseline characteristics are summarized at Table 2. Aneurysm diameter were similar both before and after matching. All the other baseline characteristic except BSA and ASI were comparable between matched groups. It was found that the ASI was higher at female group (4.12 ± 0.84 cm/m^2^ vs. 3.47 ± 0.72, *p* = 0.002) before matching and similarly after matching (4.12 ± 0.84 cm/m^2^ vs. 3.67 ± 0.56, *p* = 0.011). Compared with well-matched male population, more female patients were implanted outside the IFU (38.5% vs. 11.5%, *p* = 0.025).

**Table 2:** Baseline characteristics of the patients

Features	Unmatched cohort			Matched cohort		
	Female patients (n = 26)	Male patients (n = 223)	*p* value	Female patients (n = 26)	Male patients (n = 26)	*p* value
Age, years, median (IQR)	75 (68–83)	69 (65–74)	0.014	75 (68–83)	73 (69–77)	0.687
Diabetes mellitus	12 (46.2)	54 (24.2)	0.016	12 (46.2)	11 (42.3)	0.780
Hypertension	24 (92.3)	146 (65.5)	0.005	24 (92.3)	24 (92.3)	1.000
Hyperlipidemia	8 (30.8)	65 (29.1)	0.864	8 (30.8)	6 (23.1)	0.532
Pulmonary disease	10 (38.5)	73 (32.7)	0.558	10 (38.5)	8 (30.8%)	0.560
Renal insufficiency	0	26 (11.7)	0.087	0	2 (7.7%)	0.490
Peripheral vascular disease	1 (3.8)	21 (9.4)	0.485	1 (3.8)	2 (7.7%)	1.000
Coronary artery disease	5 (19.2)	108 (48.4)	0.005	5 (19.2)	4 (15.4%)	1.000
CABG	0	52 (23.3)	0.006	0	0	–
History of malignancy	0	14 (6.3)	0.373	0	0	–
Previous abdominal surgery	5 (19.2)	16 (7.2)	0.053	5 (19.2)	2 (7.7)	0.419
Body surface area, m^2^, mean ± SD	1.75 ± 0.24	1.88 ± 0.17	0.016	1.75 ± 0.24	1.85 ± 0.12	0.033
Aortic size index, mean ± SD	4.12 ± 0.84	3.47 ± 0.72	0.002	4.12 ± 0.84	3.67 ± 0.56	0.011
Maximum aneurysm diameter, mm, median (IQR)	62 (59–75)	60 (55–70)	0.273	62 (59–75)	63 (57–75)	0.953
Ejection fraction, % median (IQR)	55 (53–57)	55(48–58)	0.333	55 (53–57)	55 (52–60)	0.360

Intraoperative data showed that duration of procedure and fluoroscopy and amount of contrast agent used were similar between the unmatched groups (Table 3). However, regarding matched groups, duration of procedure was longer at female population (155 min vs. 127 min, *p* = 0.033).

**Table 3:** Technical details of procedures

Features	Unmatched cohort			Matched cohort		
	Female patients (n = 26)	Male patients (n = 223)	*p* value	Female patients (n = 26)	Male patients (n = 26)	*p* value
Non-IFU implantation	10 (38.5)	40 (17.9)	0.013	10 (38.5)	3 (11.5)	0.025
General anesthesia	20 (76.9)	176 (78.9)	0.419	20 (76.9)	19 (73.1)	0.919
Type of endograft						
Modular	21 (80.8)	160 (71.7)	0.329	21 (80.8)	21 (80.8)	1.000
Unibody	5 (19.2)	63 (28.3)		5 (19.2)	5 (19.2)	
Duration of procedure, min, mean ± SD	151 ± 35	141 ± 47	0.097	151 ± 35	127 ± 38	0.033
Duration of fluoroscopy, min, mean ± SD	18 ± 9	18 ± 11	0.990	18 ± 9	17 ± 7	0.845
Amount of contrast agent, ml, mean ± SD	66 ± 23	65 ± 22	0.956	66 ± 23	62 ± 19	0.641

### Outcomes and follow-up

Regarding early postoperative data, ICU stay was significantly higher in female group for unmatched groups (8 h vs. 4 h, *p* = 0.002). However, duration of ICU stay and hospital stay were comparable between matched groups (Table 4).

**Table 4:** Postoperative results

Features	Unmatched cohort			Matched cohort		
	Female patients (n = 26)	Male patients (n = 223)	*p* value	Female patients (n = 26)	Male patients (n = 26)	*p* value
Duration of ICU stay, hours, median (IQR)	8 (4–20)	4 (4–6)	0.002	8 (4–20)	4 (4–6)	0.065
Hospital stay, days, median (IQR)	2 (2–3)	2 (1–4)	0.950	2 (2–3)	2 (2–4)	0.802
Early mortality	2 (7.7)	2 (0.9)	0.055	2 (7.7)	0	0.490
Endoleak	5 (19.2)	32 (14.3)	0.558	5 (19.2)	6 (23.1)	0.734
Type 1A	0	3 (1.3)		0	0	
Type 1B	1 (3.8)	5 (2.2)		1 (3.8%)	1 (3.8)	
Type 2	3 (11.5)	15 (6.7)		3 (11.5)	3 (11.5)	
Type 3	1 (3.8)	8 (3.6)		1 (3.8)	2 (7.7)	
Secondary intervention	3 (11.5)	20 (9.0)	0.718	3 (11.5)	2 (7.7)	1.000
All-cause mortality	2 (7.7)	36 (16.1)	0.388	2 (7.7)	3 (11.5)	1.000
Follow-up, months, median (IQR)	26 (14–38)	25 (14–39)	0.950	26 (14–38)	27 (13–35)	0.722

The early mortality was about seven-fold higher in female population in the unmatched group (7.7% vs. 0.9%). However, there was no statistical difference in early mortality between the unmatched and matched groups (*p* = 0.055 for unmatched groups and *p* = 0.490 for matched groups).

Patients were followed at outpatient clinic at a median follow-up of 26 months (IQR:14–38 months). There was no difference in secondary intervention and all-cause mortality between the groups. In follow-up period, endo-leaks were seen in both groups, at a rate of 19.2% in women, and 14.3% in men (*p* = 0.558); three type 2 (11.5%), one type 3 (3.8%) and one type 1b (3.8%) endo-leaks were seen in female group. In the follow up period, late mortality was similar between groups (unmatched cohort; 7.7% vs. 16.1%, *p* = 0.388 and matched cohort; 7.7% vs.11.5%, *p* = 1.000).

## Discussion

This study showed that outcomes after EVAR in female population was comparable to those of male population.

Many possible factors such as female gender, older age, undiagnosed cardiovascular diseases, and more difficult anatomy have been proposed as a predictor for adverse events both for EVAR and open surgery [7]. Previous studies on women with AAA consist of meta-analyses and studies with series of limited patient population showing that women have higher mortality and morbidity in the early and long-term period [8–10, 13]. Although open surgery is more pronounced, mortality in women is higher in both endovascular treatment and open surgery than in men. Overall early mortality was 2.3–3.2% in women and 1.2–1.4% in men after EVAR and 5.4–8.0% in women and 2.8–4.0% in men after open surgery [8–10, 13].

As it is known, current guidelines recommend earlier attempt in women for aneurysms that are 5.5 cm in men and 5 cm in women [4]. Reasons for these differences might be the higher age of women at diagnosis and therapy, as well as genetic, hormonal, anatomical and biological differences. It has been shown that women have a higher risk of aneurysm growth rate and rupture [14]. In studies on AAA, generalization is often carried out and the onset, process and consequences of the disease in women are usually ignored [15]. Since the risk of rupture in small aneurysms is 4 times higher in women, it is necessary to intervene in aneurysms earlier [10]. Another reason for rupture in women with smaller diameters is that the initial aortic diameter is smaller according to their body size [13]. For this reason, the aortic size index can be used to consider relative expansion. Aortic size index (ASI) can be an important factor in determining the time of intervention, and should be incorporated into clinical decision making much more [16]. In our study, the BSA values of men were calculated to be 1.88 m^2^, and higher than those of women which was 1.75 m^2^. Beside this, BSA values of female patients are smaller ASI was significantly higher in female patients for matched groups. This may be the reason of more challenging anatomy and the lack of compliance with IFU criteria increasing the risk of both open surgery and EVAR. This finding may represent that the “*petit and delicate*” structure of female body needs more attention regarding to aneurysm size.

Considering technical details of procedure, duration of procedure was longer in the female patients for matched groups (151 min vs. 127 min). However, duration of fluoroscopy (18 min vs. 17 min) and amount of contrast agent used (66 ml vs. 62 ml) was comparable between groups. As technical details were not the focus of this study, further analysis have not been conducted for the longer duration of procedure. However, bilateral femoral exploration, narrower access vessels and challenging anatomy may be the reason for longer procedures.

There are several theories about the worse prognosis in women. Women remain vulnerable to all cardiovascular diseases with the decrease of estrogen levels [17]. The development of aneurysm accelerates with the disappearance of the protective effect of estrogen over atherosclerosis. Estrogen is thought to be protective against aneurysm formation by reducing the proteolytic activity [18]. It is reported that estrogen reduces the production of MMP-9 which contributes to the degradation of the vessel wall, and damages collagen, which leads to a dilatation and possible rupture of the aorta [19]. In a study of Villard et al. [18] higher expression of androgen receptors, and a lower expression of estrogen receptor were found at biopsy samples of male aorta. This may suggest that sex hormone activity could be associated with aneurysm development. Another theory is that women have a challenging anatomy such as having a hostile neck, greater angulation of the aorta, and much smaller and angulated access vessels [17].

**Fig. 3:**
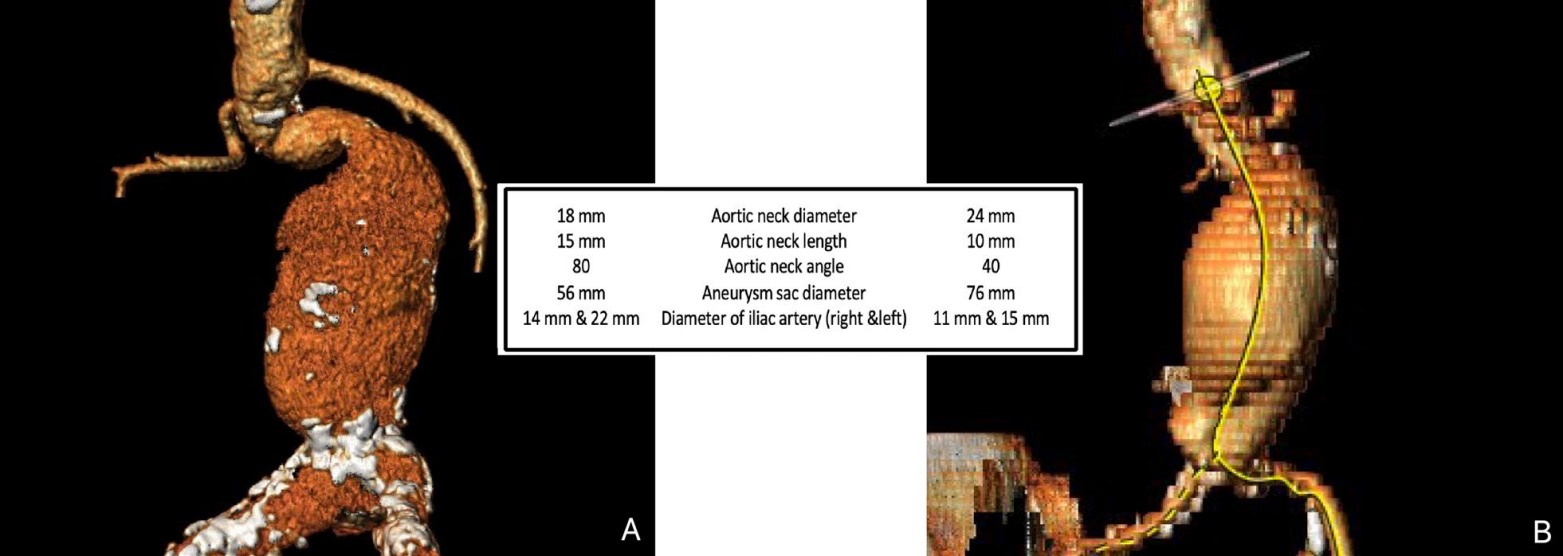
Anatomical characteristics of aneurysm of two deceased female patients. Both were IFU (instructions for use) -outside patients

Female patients had higher mortality and morbidity rates after EVAR than male patients at published papers [8, 10]. Besides, there are conflicts about the worse prognosis. Some studies have indicated comparable outcomes at female population as we have documented [20, 21]. In this study, there were 2 early mortalities among female patients who were outside IFU. Figure 3 demonstrates the anatomic structure of the deceased patients by 3D computerized tomographic images. Open surgical risks were remarkably high because of the clinical condition and comorbidities of these patients. The early and late mortality rates were similar for both unmatched and matched groups in our series. However, even though not statistically significant, early mortality was 7.7% for female patients which was remarkably high for total patient cohort (1.6%). Similarly, there are studies reporting no difference between genders in terms of mortality [14, 22, 23]. Gloviczki et al. [22] analyzed the outcomes of 934 patients (13% female) in which they have concluded that women had an increased rate of complications and re-interventions, but not a significantly higher mortality. A complete consensus has not been achieved on this subject. However, it may be logical to refer female patients to high volume cardiovascular surgery clinics offering both endovascular and open repair as suggested in current guidelines [1, 4].

Female patients occasionally treated outside the IFU due to a challenging anatomy (overall pooled data 34% for women–54% for men) [10]. Similarly, in our study, there were 10 female patients (38.5%) treated as non-IFU which was two-fold of male patients. Regarding the high risk of open surgery for these patients, endovascular procedure was performed as a first choice. However, even the disproportion at IFU, secondary intervention rates were similar between females and males (unmatched cohort; 11.5% vs. 9.0%, and matched cohort; 11.5% vs 7.7%). Higher rates of type IA endoleaks have been reported for female population [20]. However, in our study, there was no type IA endoleak in female group despite the two-fold increased non-IFU rates. New generation low profile endografts and increased experience may give better compatibility and better early results despite challenging anatomy.

### Study limitations

This study is based on a retrospective analysis of our institutional prospectively collected database. Therefore, it reveals a single-center experience and brings the major limitations of a retrospective design. The other limitation of our study is the limited female patient population similarly in other cross-sectional, single center studies and no comparison with open surgery patients for the same period. PSM should be based on treatment and control groups. However, impact of gender on the outcome was the main point of the study and PSM was performed to give a general idea about this impact. However, this is a study about the impact of genders following EVAR by reducing bias in observational studies with PSM.

## Conclusion

Non-invasive nature and less perioperative complications compared to open surgery are the pros of endovascular procedures. Challenging anatomy and subsequently treated patients outside IFU may be the reasons for higher morbidity and mortality in women. However, despite these factors female and male patients revealed equivalent early and late results. Our finding may be a reinforcement for that no matter whether they are IFU-compatible or not, EVAR should be considered as the first option in earlier intervention in women.

## Data Availability

The data generated by and used in the study is available from the corresponding author upon reasonable request.
